# R-Loops and R-Loop-Binding Proteins in Cancer Progression and Drug Resistance

**DOI:** 10.3390/ijms24087064

**Published:** 2023-04-11

**Authors:** Noha Elsakrmy, Haissi Cui

**Affiliations:** Department of Chemistry, University of Toronto, Toronto, ON M5S 3H6, Canada

**Keywords:** R-loops, RNA-DNA hybrids, cancer, chemotherapy, drug resistance, ATR, BRCA, DNA damage, DNA repair

## Abstract

R-loops are three-stranded DNA/RNA hybrids that form by the annealing of the mRNA transcript to its coding template while displacing the non-coding strand. While R-loop formation regulates physiological genomic and mitochondrial transcription and DNA damage response, imbalanced R-loop formation can be a threat to the genomic integrity of the cell. As such, R-loop formation is a double-edged sword in cancer progression, and perturbed R-loop homeostasis is observed across various malignancies. Here, we discuss the interplay between R-loops and tumor suppressors and oncogenes, with a focus on BRCA1/2 and ATR. R-loop imbalances contribute to cancer propagation and the development of chemotherapy drug resistance. We explore how R-loop formation can cause cancer cell death in response to chemotherapeutics and be used to circumvent drug resistance. As R-loop formation is tightly linked to mRNA transcription, their formation is unavoidable in cancer cells and can thus be explored in novel cancer therapeutics.

## 1. R-Loops—Physiological Appearance and Regulation

**Figure 1:**
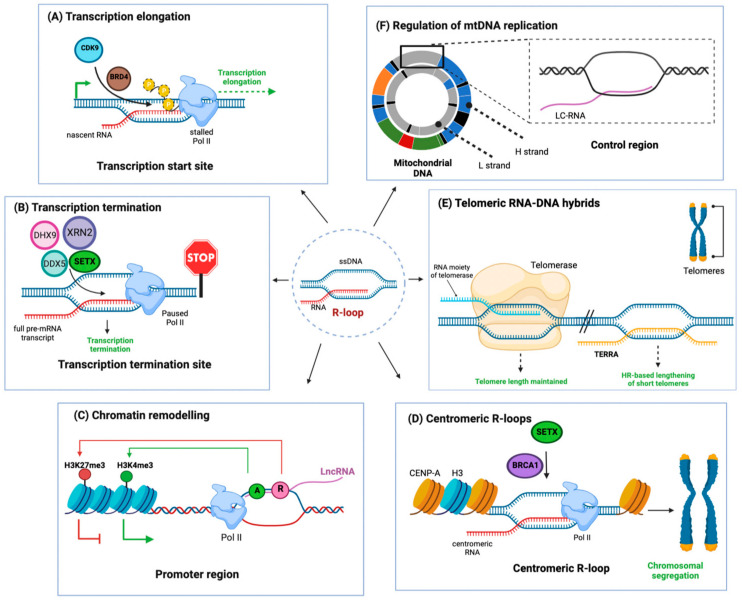
Physiological RNA-DNA hybrids serve important regulatory functions. RNA:DNA hybrids formed by a double-stranded RNA-DNA and a displaced single-stranded DNA are termed R-loops (Center). R-loops are important for several physiological cellular processes; (**A**) R-loops preferentially form at promoter sequences. Bromodomain containing protein 4 (BRD4) prevents the persistence of these R-loops by recruiting Kinases such as cyclin-dependent kinase 9 (CDK9), which phosphorylates the C-terminal domain of RNA polymerase II (Pol II), causing its transition from transcription initiation to transcription elongation. This alleviates Pol II stalling, which is a main cause for R-loop formation [1]; (**B**) At transcription termination sites, R-loop formation causes stalling of RNA polymerase II, which enforces transcription termination. Stalled Pol II is then released from the transcription site by R-loop resolving enzymes such as 5′-3′ exoribonuclease 2 (XRN2), DEAD-Box helicase 5 (DDX5), and 9 (DHX9) [1]; (**C**) R-loops can regulate gene expression: Anti-sense long non-coding RNAs (lncRNA) can reanneal to their promoter DNA. In certain loci, this will, in turn, promote activating histone modifications (A, green circle) or repressive histone modifications (R, red circle) by recruiting the corresponding enzymes. Histone modifications, in turn, enhance or repress gene expression [2,3]; (**D**) R-loops form at centromeres, where tandem repeats known as α-satellite repeats are coated with Centromeric protein A (CENP-A) and are actively transcribed by Pol II. Co-transcriptional R-loops are resolved by Senataxin (SETX), which is recruited to the R-loops by BRCA1, allowing efficient chromosomal segregation [1]; (**E**) R-loops form at telomeres through hybridization of the lncRNA TERRA (telomeric-repeat containing RNA). In critically short telomeres, this activates an alternative lengthening of telomeres through RAD51-dependent homology-directed repair (HR) [1,4]; (**F**) Mitochondria contain R-loops in their DNA, where a regulatory R-loop forms at the control region of the mtDNA by the annealing of L-strand transcripts to the control region DNA (termed L-strand control RNA, LC-RNA). Mitochondria also form a triple-strand loop structure known as D-loop—in contrast to R-loops, the third strand in D-loops is 7S DNA. Mitochondrial R-loop and D-loop are important for regulation of mtDNA replication [8]. ssDNA: single-stranded DNA; L-strand: light strand; H-strand: heavy strand; LC-RNA: L-strand control RNA; CDK9 (kinase): cyclin-dependent kinase 9; BRD4 (chromatin reader): Bromodomain-containing protein 4; Pol II (DNA transcription): RNA polymerase II; DHX9 (helicase): DEAD box helicase 9; DDX5 (helicase): DEAD Box helicase 5; SETX: Senataxin (helicase). H3: histone variant Histone 3; XRN2 (cleaves RNA): 5′-3′ exoribonuclease 2; CENP-A (centromeric nucleosome protein): Histone H3-like centromeric protein A; TERRA (long non-coding RNA): Telomeric-repeat-containing RNA; BRCA1 (DNA repair); Breast cancer type 1 susceptibility gene.

R-loops are nucleic acid structures composed of a DNA–RNA double strand and a displaced single-stranded (ss) DNA. During transcription, the nascent mRNA transcript can anneal to its template DNA strand, which gives rise to these DNA–RNA hybrids [1]. In addition, R-loops can form through the pairing of non-coding RNAs with chromosomal DNA [1]. While transient by nature, R-loops preferentially form in regions at or in close proximity to transcription initiation (Figure 1A) and termination sites (Figure 1B) [1]. R-loops mediate transcription termination in gene-dense genomic areas (Figure 1C), where the formation of R-loop signals for RNA Polymerase II (Pol II) to pause and disengage [2,3]. Disruption of the RNA splicing machinery can also cause increased R-loop formation, as the unspliced mRNA is retained close to its DNA template [4].

R-loops have regulatory functions at pericentromeric (Figure 1D) and telomeric regions (Figure 1E), which are both transcribed by Pol II [1]. Telomeres are protected by R-loops, which form between telomeric repeats and long non-coding RNAs [5]. More specific roles of genomic R-loops include transcription regulation and immunoglobulin class switch recombination [3,6,7]. In addition to the genomic DNA, R-loops are also crucial regulatory sequences in mitochondrial DNA (mtDNA), where they prime mtDNA replication (Figure 1F) [8]. R-loops are found throughout all domains of life—in bacteria and archaea, their formation is part of an internal defense mechanism against viruses, where CRISPR type I triggers R-loop formation and subsequent DNA degradation [9,10]. Their regulated appearance in the mammalian genome indicates that they were co-opted for regulatory activities, in addition to their formation as a seemingly unavoidable side-product of RNA transcription.

In addition to R-loops generated during Pol II activity during the transcription of mRNA, R-loops also arise from RNA transcription through RNA polymerase I and III (Pol I and Pol III, respectively) [11,12]. While Pol I generates rRNA [13], which form the core of ribosomes, which in turn translate mRNAs into proteins, Pol III generates 5S ribosomal RNA as well as predominantly non-coding RNAs, such as tRNAs, snoRNAs, spliceosomal, and Y RNAs [14]. As many of these non-coding RNAs are heavily transcribed, they are prone to R-loop formation, which has been localized to rRNAs, tRNAs, and retrotransposons [11,12]. Interestingly, R-loops at tRNA genes are more resistant to RNAse H treatment [12] and produce both sense- and anti-sense-paired R-loops, reflective of the tRNA’s cloverleaf fold, which is formed through intramolecular complementary regions [15]. In consequence, these highly transcribed areas are especially vulnerable to DNA damage caused by the prolonged persistence of R-loops.

**Figure 2:**
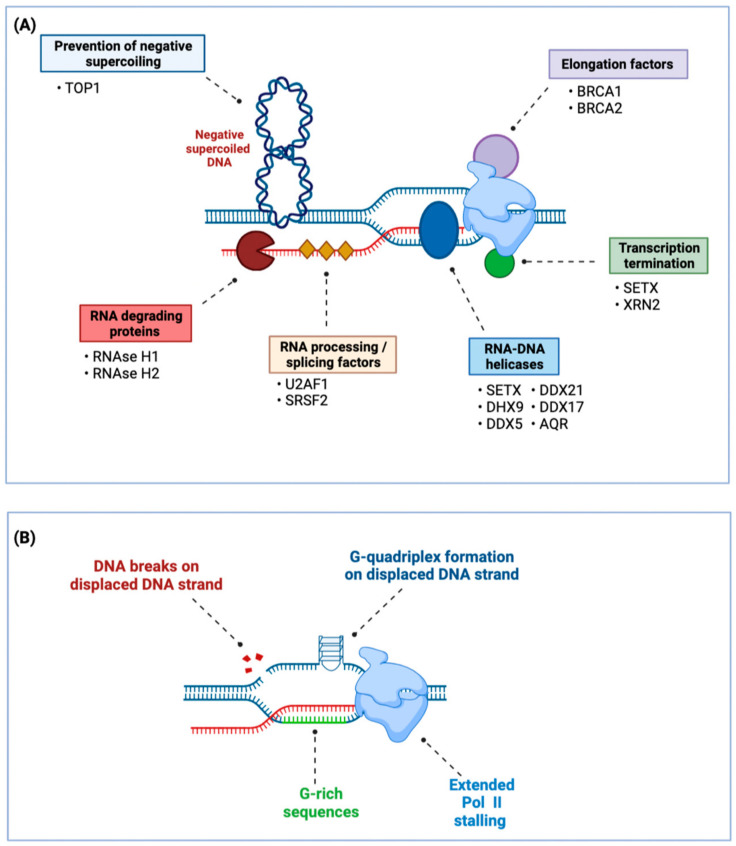
R-loop resolution and formation in the cell. (**A**) Prevention and resolution of R-loops. TOP1 unwinds negative supercoiling resulting from strand unwinding during transcription and thereby prevents mRNA from annealing to unwound DNA, thereby preventing R-loop formation [29]. RNA processing and/or splicing factors such as SRSF2 and U2AF1 prevent R-loop formation by coating of the nascent mRNA transcript and thereby preventing annealing to the template strand [30,31]. BRCA1 and BRCA2 prevent Pol II stalling and promote transcription elongation by acting as elongation factors, thereby preventing R-loop formation by ensuring processive transcription [32,33,34]. SETX and XRN2 signal Pol II termination, which prevents R-loop formation due to prolonged Pol II stalling [19]. Once formed, aberrant R-loops can be resolved through degradation of the RNA component by RNAse H1 or H2, which specifically degrade RNA-DNA hybrids. R-loops can also be resolved by unwinding the RNA-DNA hybrid through the helicase activity of SETX, DHX9, DDX5, DDX17, DDX21, and AQR [1,20,21]; (**B**) Physiological and pathological processes that promote R-loop formation. R-loops preferentially form when Pol II is stalling. This can be caused by DNA damage, structural hindrance through G-quadruplex formation, or tighter base-pairing in GC-rich sequences [1]. TOP1 (transient breaking and rejoining of single-stranded DNA): Topoisomerase I; SRSF2 (RNA-binding protein): serine and arginine rich splicing factor 2; U2AF1 (splicing factor): U2 small nuclear RNA auxiliary factor 1; SETX (helicase): Senataxin; XRN2 (DNA incision): 5′-3′ exoribonuclease 2; DHX9 (helicase): DEAD box helicase 9; DDX5 (helicase): DEAD Box helicase 5; DDX17 (helicase): DEAD Box helicase 17; DDX21 (helicase): DEAD Box helicase 21; AQR (pre-mRNA splicing): RNA helicase aquarius; Pol II (DNA transcription): RNA polymerase II.

As collisions between R-loops and the replication fork lead to DNA damage, R-loop formation and resolution must be carefully controlled, particularly during replication in the S phase. These collisions can cause single-strand and double-strand DNA breaks and threaten genomic stability [16]. Factors regulating R-loops (Figure 2A) include RNase H, which degrades the annealed RNA transcript in R-loops, thereby resolving these structures. RNA-DNA mismatches prevent RNAse H1-dependent R-loop resolution [17], and inactive RNAse H can be used to map R-loops [18]. In addition to specific degradation of the RNA strand, the helicase Senataxin (SETX) unwinds R-loops and enables subsequent cleavage [19]. Other helicases, such as RNA helicase aquarius (AQR) and the DEAD Box helicase also resolve R-loops [1,5,9,17,20,21]. Similarly, topoisomerase I resolves supercoils, which prevents the formation of transcription-induced R-loops. These have been extensively reviewed elsewhere [8,16,22,23]. Epigenetic modifications also influence R-loop formation (Figure 2B). Open, more accessible chromatin is more susceptible to DNA:RNA hybridization [24], and m6A modifications of the RNA strand stabilizes R-loops [25].

R-loop perturbations are recorded in several auto- and neuroinflammatory disorders (reviewed in [26,27]). In addition to their contribution to neuropathology, R-loops play a critical role in cancer through their complex interplay with known tumor suppressors and oncogenes, as well as due to their central role in maintaining genome stability. Building on previous excellent reviews of the topic [1,21,28], we discuss the control of R-loop formation, persistence, and the response to R-loops as a double-edged sword for cancer cells: During tumorigenesis, a dysregulation of proteins that recognize and resolve R-loop and the resulting DNA damage accelerates the mutational burden, enabling cancer progression and promoting resistance. However, an excess of R-loops destabilizes the genome, causing senescence in cancer cells and failures in cell division. Elucidating the molecular details of R-loop mitigation and their dysregulation in cancer sheds new light on key players in tumor suppression and highlights new therapeutic avenues by targeting an unavoidable side-product of mRNA transcription and cell proliferation.

## 2. Pro-Tumorigenic Effects of R-Loops in BRCA1/2 Mutant Cancers

**Figure 3:**
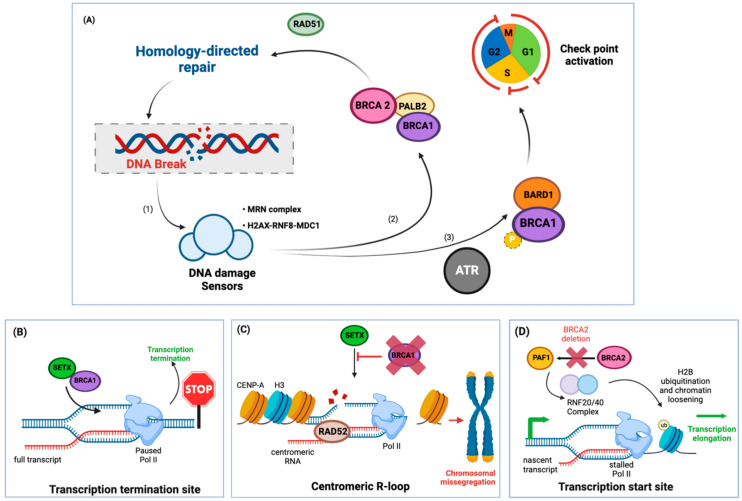
BRCA1/2 tumor suppressors protect the genome against DNA damage by resolving R-loops. (**A**) (1) DNA damage is detected by different DNA damage sensors such as the MRN complex, which senses double-stranded breaks, or the H2AX-RNF8-MDC1 complex, which recognizes histone ubiquitylation, which are installed proximal to sites of DNA damage [37]. (2) Upon its recruitment by DNA damage sensors, BRCA1 directly binds various damage response mediators such as Topoisomerase II binding protein 1 (TOPBP1) or BRCA1–partner and localizer of BRCA2 (PALB2) to form a complex with BRCA2 and initiate RAD51-dependent homologous recombination [37]. (3) ATR- or ATM-dependent phosphorylation of BRCA1, while it is in complex with the BRCA1-associated RING domain protein 1 (BARD1), induces cell cycle arrest to allow time for DNA repair; [37] (**B**) BRCA1 recruits senataxin (SETX) to unwind R-loops and enables transcription termination and the release of Pol II (RNA polymerase II) [32]. Loss of BRCA1 through mutations in cancer causes the accumulation of R-loops and can accelerate DNA damage; (**C**) Loss of BRCA1 activity impairs the recruitment of senataxin to centromeric R-loops, leading to unresolved R-loops and the subsequent accumulation of Rad52-dependent hyper-recombination, centromere breakage, micronuclei formation, and chromosomal missegregation [38]; (**D**) BRCA2 mediates the release of stalled Pol II through recruitment of PAF1 (RNA polymerase II-associated factor 1) [34,39]. In turn, PAF1 recruits the RNF20/40 E3 ubiquitin ligase complex to ubiquitinate histone subunit 2B. This modification enables histone dissociation and Pol II access to the downstream sequence, resolving Pol II stalling. Disruption of this process by BRCA2 deletion or inactivation, as often found in cancer, results in R-loop and Pol II accumulation at promoter proximal pause sits. ATM (DNA damage sensor): Ataxia telangiectasia mutated. SETX: Senataxin (helicase). H3: histone variant Histone 3. Cell cycle: G1: growth phase 1; S: DNA synthesis phase; G2: growth phase 2; M: mitosis.

Breast cancer genes 1 and 2 (BRCA1 and BRCA2) are tumor suppressor genes, and mutations in either strongly increase the risk of the carrier towards developing breast and ovarian cancer [35]. Despite the similar name, which is owned by their risk profile in developing breast cancer, their molecular function is distinct. *BRCA1/2* mutations increase the risk of breast cancer almost 6-fold while the risk for ovarian cancer rises about 25-fold over a patient’s lifetime [35,36]. BRCA1 and 2 are both central to protecting the integrity of the genome [37] and function in homologous recombination, a DNA repair program (Figure 3A). As R-loops can cause double-strand breaks if unresolved, BRCA1 and BRCA2 play crucial roles in mediating the physiological functions of R-loops as well as responding to potential harmful damage caused by their accumulation.

BRCA1 and BRCA2 function is tied to RAD51, a DNA binding protein, which modulates the replication fork during DNA synthesis, and also stabilizes R-loops that are formed in trans [40]. Specifically, BRCA1 is a multidomain protein that contains, among others, E3 ligase activity [41] and a phospho-protein binding domain (BRCT) [42]. Through these, BRCA1 undergoes a series of interactions that links it to both the sensing of DNA double-strand breaks and the initiation of DNA damage repair [37,43]. BRCA1 also directly binds various damage response mediators, such as Topoisomerase II binding protein1 (TOPBP1), to initiate RAD51-dependent homologous recombination or impose cell cycle progression, among other functions [37]. BRCA2 also interacts with RAD51 and mediates the assembly of RAD51 complexes at the site of DNA damage [44,45] and stabilizes RAD51-single-stranded DNA filaments during DNA replication [46,47]. In addition to guiding the DNA repair machinery, BRCA1/2 perform regulatory roles in transcription regulation and chromatin structure rearrangements, many of which have recently been shown to be R-loop-dependent. As classical tumor suppressors, BRCA1/2 mutations that are disease-associated are predominantly loss-of-function, with 70% of BRCA1 mutations leading to a loss of full-length protein [48].

### 2.1. R-Loops at the Site of Active Transcription in BRCA1/2 Mutant Cancers

BRCA1 regulates transcription by binding to physiologically formed R-loops at both transcription initiation and transcription termination sites [32,33]. Planned R-loop formation is a prerequisite to terminate transcription as it signals the release of RNA polymerase II [32]. When transcription terminates, BRCA1 is necessary for the proper assembly of the termination machinery (Figure 3B). Here, BRCA1 binds R-loops and recruits the R-loop-resolving protein Senataxin [32]. Knockdown of either BRCA1 or Senataxin triggers R-loop persistence and induces single-stranded breaks at these sites [32]. In consequence, *BRCA1*-mutated breast cancer cells harbor significantly higher insertion-deletion mutations around known transcription termination sites, suggesting that BRCA1-directed resolution of R-loops halts tumorigenesis [32] (reviewed in [49,50]).

Furthermore, BRCA1 guards the centromeric regions of chromosomes by interacting with Senataxin and preventing toxic R-loop build-up (Figure 3C) [38]. Centromeric DNA is characterized by repetitive sequences, which are coated with a histone variant known as CENP-A [51]. These sequences are actively transcribed throughout the cell cycle and are thereby susceptible to co-transcriptional R-loop formation. BRCA1 recruits Senataxin to sites of centromeric R-loop build-ups, promotes their resolution, and suppresses double-stranded breaks [38]. In contrast, in BRCA1 mutant cells, R-loops accumulate, causing double-strand breaks, CENP-A depletion, and micronuclei formation, which is indicative of defective chromosomal segregation [38].

BRCA2 prevents R-loop accumulation, and in consequence, its depletion causes increased build-up of R-loops [39]. These R-loops accumulated particularly at promoter proximal pause sites [34], which are Pol II regulatory sequences close to transcription initiation sites (Figure 3D) [52]: after Pol II initiates transcription, it stalls on the transcribed sequence waiting for a transcription elongation signal [53] and prolonged stalling can lead to unscheduled R-loop formation [34]. BRCA2, in turn, can recruit Pol-II-associated factor 1 (PAF1) to these promoter proximal pause sites to enable transcription elongation [34], thereby resolving R-loops. In consequence, *BRCA2* deletion induces Pol II stalling and build-up of R-loops at promoter proximal pause sites, leading to double-strand breaks [34].

### 2.2. R-Loop-Driven Control of Luminal Differentiation

In addition to regulating transcriptional activity, BRCA1 promotes the maturation and differentiation of breast luminal tissue, and impaired differentiation is a phenotype of *BRCA1* mutations in breast cancer cells [54,55]. This is regulated partially through R-loop structures, which accumulate preferentially in luminal epithelial cells in contrast to other breast tissues of *BRCA1* mutation carriers [56]. RNAse H1 overexpression, which degrades the RNA strand of the R-loop, led to their resolution and restored cell differentiation in *BRCA1* mutated cells [56]. This study suggests that aberrant R-loop formation and the subsequent disruption of cell differentiation in breast luminal tissue is one of the causative mechanisms for breast cancer and one of the tissue-specific consequences of BRCA1 mutations.

### 2.3. The Role of R-Loops in Telomere Maintenance

Telomere maintenance is a prerequisite for cancer cells to proliferate indefinitely, and failure thereof leads to senescence and cell death [57]. As such, upregulation of telomerase activity or homologous recombination-based “alternative lengthening of telomeres” (ALT) is observed in almost all tumor cells [58]. Interestingly, in both cases, R-loop-induced telomeric instability is observed. Telomeric-repeat-containing RNA (TERRA), a class of long non-coding RNAs transcribed from chromosomal ends that regulate telomerase activity, forms R-loops with the C-rich telomeric strand in a physiological process ensuring telomere maintenance [5,59]. Deletion of *BRCA1* or *BRCA2* induces the upregulation of TERRA RNA in cancer cells [60,61]. In turn, excess TERRA is associated with telomeric R-loops and now causes telomere instability in *BRCA1* deficient cells [60].

Telomeric R-loops are further regulated through the RNA editing enzyme ADAR1 [62]. ADAR1 converts A-C mismatches at telomeres, which enables RNAse H2 to resolve telomeric R-loops, thereby maintaining telomere integrity and promoting tumor cell proliferation [62]. In line with this, telomerase-negative tumors that depend on the alternative lengthening of telomeres are highly dependent on R-loop resolution through RNAse H1 [5]. Upregulation and downregulation of RNAse H1 specifically compromised telomere stability in ALT tumor cells but not in telomerase-positive cells [5]. Taken together, telomeric R-loops sustain the indefinite proliferative characteristics of cancer cells and facilitate their propagation.

### 2.4. R-Loops in Other Breast Cancers

In addition to BRCA1/2 mutations, high exposure to estrogen also increases the risk of breast cancer. R-loop abnormalities cause DNA damage in the promoter sequences of estrogen-responsive genes, causing double-strand breaks and genomic instability [63]. Induction of estrogen-responsive genes led to the rapid accumulation of R-loops, particularly in the vicinity of estrogen-responsive genes, which in turn caused double-stranded breaks and increased mutation frequency [63]. Given that *BRCA1*/*2*-mutation also lead to increased and unresolved R-loop formation, targeting the R-loop modulating machinery may be beneficial in breast cancer.

### 2.5. BRCA2 and R-Loops in the Mitochondrial Genome

RNA/DNA hybrid structures are not only found in nuclear DNA. Rather, planned R-loop formation serves regulatory functions in the mitochondrial genome (mtDNA) as well (reviewed recently [64]). Indeed, mtDNA replication is initiated by the reannealing of the small RNA transcripts from the promoter region of the mitochondrial light chain to its coding strand, forming the D-loop, which functions as a primer for DNA polymerase γ [65]. Nevertheless, unplanned R-loop formation is likewise unfavored and is regulated by the mitochondrial degradosome [66]. This conserved RNA surveillance complex is composed of the RNA/DNA helicase SUV3, the poly (A) polymerase, and exoribonuclease PNPase, which degrades dsRNA and DNA/RNA hybrids to maintain mtDNA replication and integrity [67]. Similar to the nucleus, the R-loop specific RNAse H1 is also found in the mitochondria, where it actively degrades the RNA component of RNA-DNA hybrids [68].

mtDNA mutations are linked to tumor growth and metastasis through the excess generation of reactive oxygen species and mitochondrial dysfunction [69]. As a result, some clinically used anticancer drugs rely on the induction of reactive oxygen species for their cytotoxicity. BRCA1 regulates oxidative stress by modulating gene expression, among other mechanisms [70]. Interestingly, BRCA2 inactivation causes mitochondrial R-loop accumulation through ROS by indirectly impairing the recruitment and binding of mitochondrial RNAse H1, causing impaired transcription initiation and DNA replication [71]. This is mediated on a molecular level through increased 8-oxoguanine base adduct formation during oxidative stress, which is usually resolved through base excision repair [71]. If this repair process is disrupted, the resulting mismatch leads to an accumulation of R-loops [71]. Untangling the abnormalities relating to mitochondrial R-loop formation can provide important insights into inducing mitochondrial-dependent cytotoxicity in cancer cells.

Overall, BRCA1 and BRCA2 mediate R-loop formation and resolution through several mechanisms, as a controlled byproduct in both transcription initiation and termination, as well as at telomeric sites regulating chromosomal stability. In addition to BRCA1/2, other breast cancer risk-causing factors, such as estrogen receptor signaling and reactive oxygen species, can cause the accumulation of R-loops and/or hinder their resolution. R-loop formation in BRCA1/2 deficient cells causes genomic instability, which increases mutation rates and, ultimately, cancer risk.

## 3. R-Loop Sensing through ATR and the Activation of DNA Repair Pathways

The ataxia telangiectasia and rad3-related (ATR) protein is a master regulator of DNA damage response, particularly upon replication stress. ATR is a serine/threonine kinase that is activated in response to single-stranded DNA, which is a component of R-loops. Once activated, ATR stabilizes replication forks and mediates DNA repair before cell division. As such, selective inhibition of ATR causes extensive DNA damage, which accumulates throughout cell cycle progression, resulting in mitotic collapse and cell death. This makes ATR an attractive target for cancer therapies [72]. Indeed, ATR inhibitors are currently in clinical trials for the treatment of advanced solid tumors [73,74,75,76]. Interestingly, ATR acts partially through R-loops.

### 3.1. ATR-Activation through R-Loops

**Figure 4:**
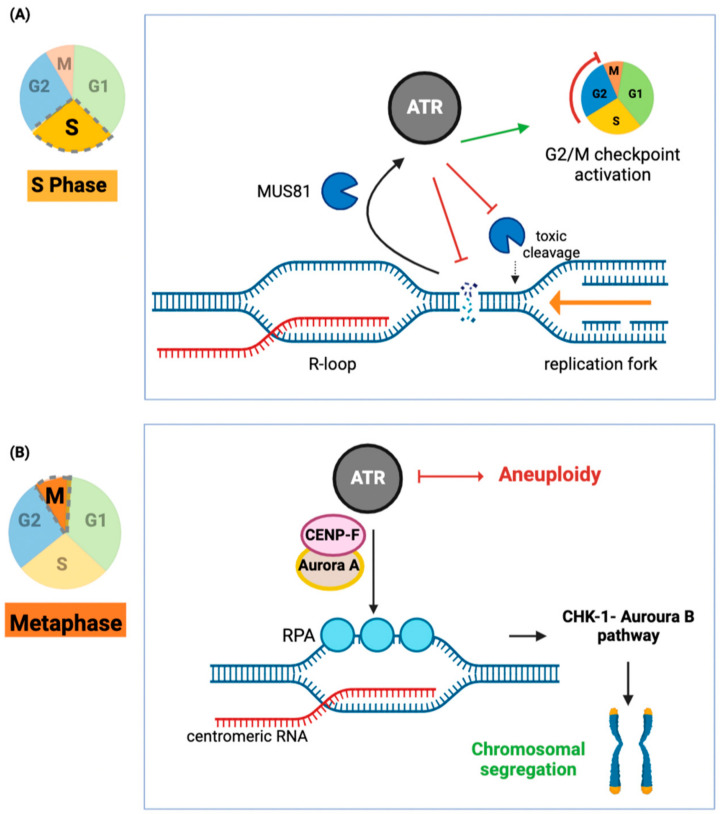
ATR protects the genome against DNA damage upon R-loop formation and ensures faithful chromosomal segregation. (**A**) During the S phase (DNA synthesis), unplanned R-loop formation signals the recruitment of ATR by the endonuclease MUS81 [77]. This activates a DNA damage response and G2/M cell cycle arrest, allowing time for fork recovery and preventing excessive double-strand break formation. ATR also signals a negative feedback response to MUS81 to prevent fork degradation mediated by MUS81’s endonuclease activity; (**B**) During cell division, ATR is recruited to centromeric R-loops, which are coated with replication protein A (RPA) by Aurora-A, a serine/threonine-protein kinase, and the microtubule-binding protein centromere protein F (CENP-F). This enables efficient chromosomal segregation through activation of Chk1-Auroura-B pathway. In consequence, inhibition of ATR results in improper chromosomal segregation and aneuploidy [79]. ATR (DNA damage sensor): Ataxia telangiectasia-related. Cell cycle: G1: growth phase 1; S: DNA synthesis phase; G2: growth phase 2; M: mitosis.

ATR protects cells from R-loop-induced DNA damage [77]. In dividing cells, the accumulation of genomic R-loops impedes the replication machinery by physically hindering replication forks, leading to replication collision events and subsequent DNA damage (Figure 4A). ATR is activated by R-loops and responds by cell cycle arrest to suppress DNA damage [77]. The activation of ATR is initiated by the recruitment of the endonuclease MUS81 to the replication fork [77]. There, MUS81 promotes single-stranded DNA formation in dependency on R-loops, which in turn activates ATR [77]. Indeed, inhibition of MUS81 in gastric cancer cells induces cell death in an ATR-Chk1-dependent mechanism [78], which could be due to excessive R-loop mediated DNA damage.

In addition, ATR guards chromosomal segregation via an R-loop-dependent mechanism [79] (Figure 4B). During mitosis, ATR is activated at centromeres through the formation of R-loops and the subsequent presence of displaced, single-stranded DNA: this single-stranded DNA is then bound by replication protein A (RPA), which in turn activates ATR [67]. This pathway is again distinct from DNA damage-induced ATR activation and is indeed necessary for mitosis [79]. R-loop-dependent ATR-activation thereby fulfills two needs in the cell, one is to ensure faithful replication, and the other is to initiate DNA damage repair [79].

### 3.2. ATR Inhibition Sensitizes Cancer Cells to R-Loop-Induced DNA Damage

**Figure 5:**
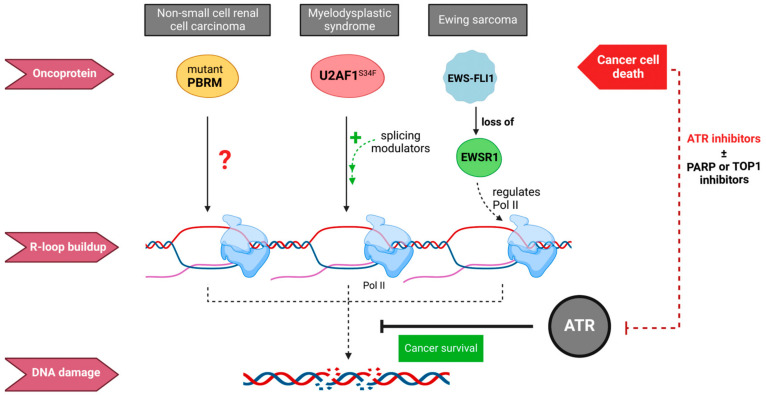
ATR inhibition induces cancer cell death through accumulation of R-loops. Non-small cell renal cell carcinoma caused by PBRM mutations [81], myelodysplastic syndrome caused by a splicing defect in U2AF1 [30], and Ewing sarcoma caused by EWS-FLI1 oncogene fusion [80] and subsequent loss of intact EWSR1 all display increased R-loop levels. Mechanistic details are unclear in PBRM mutations, hence the question mark. Splicing modulators further increase R-loops accumulation in myelodysplastic syndrome cells. A loss of the EWSR1 regulatory effect on Pol II might be causal in Ewing Sarcoma. In all three cancer cells, ATR controls the level of the resulting genomic instability to keep cancer cells viable. In consequence, ATR inhibition alone or in combination with therapies causing further DNA damage can induce critical R-loop accrual and thereby lead to cancer cell death. This mechanism, built on the lethal build-up of R-loops is explored in cancer therapy. ATR (DNA damage sensor): Ataxia telangiectasia-related; PARP (DNA repair protein): poly(ADP-ribo)polymerase; TOP1 (transient breaking and rejoining of single-stranded DNA): topoisomerase 1; PBRM (chromatin remodeling): Protein polybromo-1; U2AF1 (splicing factor): U2 small nuclear RNA auxiliary factor 1; EWSR1 (transcriptional repressor): Ewing Sarcoma protein 1; Pol II (DNA transcription): RNA polymerase II.

In turn, ATR inhibition is particularly toxic to cancer cells with an accumulation of R-loops [30,80,81] (Figure 5). An example is Ewing Sarcoma, a pediatric bone and soft tissue cancer. In Ewing sarcoma, chromosomal translocations give rise to the fusion protein Ewing sarcoma EWS-Fli1 (type 1) oncogene (EWSR-FLI1) [80]. Fusion to FLI1 disrupts the function of the RNA processing enzyme Ewing Sarcoma Protein 1 (EWSR1) and subsequent transcriptional dysregulation [80,82]. As EWSR1 and EWSR-FL1 both interact with the transcription and splicing machinery, an accumulation of R-loops is likely, and indeed, R-loop levels are 4-fold increased [80]. This led to increased ATR activation and caused the sequestration of BRCA1, mimicking the mutational loss of BRCA1 [80]. In line with these findings, Ewing sarcomas are especially susceptible to damage induced by chemotherapy drugs, many of which accelerate genomic instability upon increased R-loop formation.

R-loops also play a fascinating role in regulating ribosomal RNA generation, where Pol II generates R-loops at sequences flanking rRNAs, which are transcribed by Pol I, to shield surrounding genes from generating non-coding RNAs [83]. Disruption of this process, for example, through loss of Senataxin, disrupts nuclear organization and causes aberrations reminiscent of those found in Ewing Sarcoma through disrupting nucleoli [83]. In addition to Senataxin, ‘Eukaryotic translation initiation factor 4A’ (EIF4A3) also unwinds, specifically nucleolar R-loops [84]. EIF4A3 is a helicase involved in RNA splicing, which locates in exon-junction complexes and, as such, mediates nonsense-mediated decay. In addition, it also clears R-loops in the nucleoli [84], which can be caused by Pol I during rRNA transcription or in the above-mentioned mechanism through Pol II. This connection has been recently reviewed in the context of its interplay with p53, one of the most famous tumor suppressors and the so-called guardian of the genome [85].

Likewise, the most common myeloid malignancy in adults, myelodysplastic syndrome, can be caused by mutations in spliceosome genes (including SRSF2 and U2AF1), which occur early in the disease, supporting their role in disease pathology and progression. Increased R-loop formation upon mutations in different spliceosomal proteins has been proposed as the unifying factor causing this disease but, surprisingly, seems to be independent of the regulation of splicing [31]. Increased RNA binding in SRSF2 mutations, together with the weakened ability to release a transcription elongation factor, led to stalling of Pol II and, in consequence, increased R-loop formation [31]. HeLa cells expressing a mutant U2AF1 accumulate R-loops [30]. The increase in R-loops, in turn, causes ATR activation, and similar to Ewing sarcoma, cells expressing mutant U2AF are sensitive to ATR inhibition [30].

A similar observation is also reported in clear cell renal cell carcinoma, which comprises around 90% of all renal cell carcinoma [86]. The tumor suppressor protein polybromo-1 (PBRM1) is a chromatin remodeling protein mutated in nearly 40% of clear cell renal cell carcinoma, causing genomic and chromosomal instability [87]. PBRM1 facilitates the re-priming of stalled replication forks, and its deficiency, in turn, leads to elevated levels of R-loops in clear cell renal cell carcinoma [81]. This also correlates with enhanced sensitivity to ATR inhibition as ATR prevents R-loop-dependent DNA damage [81].

Despite the mutational heterogeneity of Ewing sarcoma, myelodysplastic syndrome, and clear cell renal cell carcinoma, overexpression of the R-loop resolving RNAse H1 successfully restores resistance to ATR inhibition in all three cancers discussed above. This supports the notion that sensitivity to ATR inhibition is rooted in an increased R-loop burden and R-loop-induced genomic instability [30,80,81].

## 4. Anticancer Effects of R-Loops and Their Exploitation for Therapy

As R-loop formation can cause DNA damage if unresolved, perturbations of R-loop homeostasis could be a viable strategy to induce cancer lethality and restore sensitivity to anticancer treatments. We have previously described mechanisms through which R-loops can be oncogenic by causing increased mutation rates upon the loss of tumor suppressors. However, an excess of double-strand breaks, mutational burden, and the disruption of chromosomal separation, all mediated through R-loops, are decisively anti-tumorigenic and will cause cancer cell death.

### 4.1. Inhibition of the DNA Damage Response through R-Loops

In addition to the aforementioned BRCA1/2 and ATR-mediated DNA damage response pathways, the PARP (poly(ADP-ribose) polymerase) family of proteins are central mediators of the DNA damage response. PARP also responds to single-strand DNA breaks, which can occur at the site of R-loop formation. In consequence, PARP inhibition has been explored as an anticancer strategy to cause synthetic lethality in cells with defective homologous recombination repair.

This is of particular importance in *BRCA1/2*-driven breast and ovarian cancer cells. The E3 ubiquitin ligase RNF168 is a double-strand break responder that promotes non-homologous end joining of double-strand breaks by recruiting BRCA1 and other repair factors to sites of damage, including R-loops [88]. There, RNF168 directly ubiquitylates the R-loop helicase DHX9, which causes its recruitment to R-loops and their subsequent resolution [89]. Decreased expression or loss of RNF168 correlated with a lower incidence of tumors in *BRCA1/RNF168* double knockout mice as well as a better survival outcome in patients with homologous repair deficient tumors [89].

BRD4 inhibition also downregulates Topoisomerase II binding protein 1, a DNA damage response protein, which inhibits activation of the ATR pathway [90]. As a result, ATR does not induce cell cycle arrest, leading to proliferation despite severe DNA damage and causing replication stress. Cancer cells treated with BRD4 inhibitors subsequently suffer not only damage arising from double-strand breaks and R-loop accrual at a subset of BRD4-controlled genes but also mitotic catastrophe, all leading to cell death [90,91].

### 4.2. Cancers with Elevated R-Loop Formation Are Susceptible to DNA Damage

Chemosensitivity and cell death through R-loops are especially relevant in cancer cells that already harbor intrinsically elevated R-loop levels. As discussed earlier, Ewing sarcoma cells contain an increased burden of R-loops due to the mechanism underlying their tumorigenicity and are therefore highly sensitive to ATR inhibition [80]. In addition to ATR inhibitors, Ewing sarcoma cells are quite vulnerable to transcription blockade through Topoisomerase and PARP inhibitors as well [80].

Elevated R-loop levels also potentiate the cytotoxicity of anticancer drugs in triple-negative breast cancer [92]. This aggressive breast cancer subtype is associated with poor prognosis and high rates of chemotherapy and radiotherapy resistance [93]. In a subset of triple-negative breast cancer cells, the double-strand break repair protein MRE11 is mutated [94]. Physiologically, Mre11 senses transcription-induced double-strand breaks and initiates a DNA damage response to defy genomic instability. However, breast cancer cells that are deficient in Mre11 accumulate R-loops and, in turn, R-loop-dependent DNA damage [92]. This increases their vulnerability to further DNA damage induced by PARP and ATR inhibitors [92].

Another malignancy with increased R-loop formation is Embryonal Tumor with Multilayered Rosettes (ETMR), which are aggressive tumors that occur in the brain. Comparison between ETMR and other brain tumors, as well as healthy brain tissue, suggests that mutations induced by R-loops are causative [95]. Mutational patterns are similar to those found in Ewing Sarcoma, and an increased number of R-loops were found surrounding the most common mutation site [95]. R-loop sites also coincided with mutation and breakpoint hot spots in these tumors [95]. The resulting genomic instability renders ETMR sensitive to DNA damaging agents, and administration of PARP and TOP1 inhibitors results in the synergistic killing of ETMR cells resistant to conventional platinum therapy [95]. Interestingly, the most common amplification and fusion event predisposing patients to ETMR affects a microRNA (miRNA) cluster, suggesting a connection between miRNA processing and R-loop formation [95]. Drosha, a key enzyme in miRNA processing, has both been previously associated with the formation of R-loops as it stabilizes RNA-DNA hybrids at DNA break sites and recruits repair factors [96]. In plants, R-loops arise at miRNA loci, initiating co-transcriptional processing of miRNAs, directly linking miRNAs, and stabilizing R-loop formation [97].

### 4.3. Induction of R-Loop Formation in Anticancer Therapy

Another approach to induce R-loop-dependent anticancer effects is by impeding the transcription machinery. As described earlier, Pol II stalls on the transcribed sequence, waiting for a transcription elongation signal [53]. This is signaled by the phosphorylation of a serine in the C-terminal domain of Pol II, allowing Pol II release and transcription elongation [98]. One of these elongation signals is triggered by Bromodomain-containing protein 4 (BRD4), which directly interacts with cyclin-dependent kinase 9, which in turn phosphorylates Pol II [99]. Due to its pro-oncogenic role in leukemia, BRD4 inhibitors have been tested and show promising pre-clinical results [100,101,102]. As BRD4 inhibitors induce cancer cell death by promoting stalling of Pol II, their mechanism of action also promotes the subsequent annealing of the transcribed pre-mRNA strand to its template, hence forming R-loops [91]. Accrual of transcriptional R-loops in the S phase results in collisions between the transcription and the replication apparatus and causes double-strand breaks [91].

JTE-607, a cytokine inhibitor with promising outcomes in the treatment of acute myeloid leukemia and lymphoma [103,104], was recently shown to perturb R-loop homeostasis. In an interesting mode of action, JTE-607 inhibits pre-mRNA release during transcription, leading to elevated R-loop levels [105]. This halts tumor growth in mouse xenografts and induces apoptosis [105], suggesting that JTE-607 may be effective in the treatment of tumors with increased R-loop levels.

**Figure 6:**
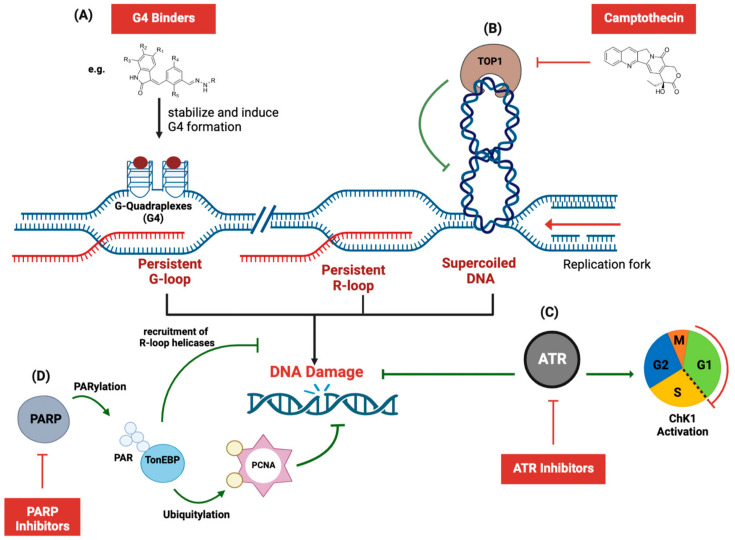
R-loop-induced genomic instability as a mechanism of action of DNA damaging agents explored in cancer therapy. (**A**) G4 binders such as Monohydrazone-derivatives stabilize G-loops (R-loops with G4 structures on displaced DNA) and thereby induce genomic instability [109,110]; (**B**) Supercoiling of the DNA can cause R-loop built-up and is rescued by the unwinding activity of topoisomerase I [29]. Inhibition of Topoisomerase, in turn, causes R-loop accrual and DNA damage; (**C**) R-loop induced double-strand break formation and subsequent DNA damage can also be sensed and repaired through the DNA damage response mediated by ATR-Chk1 activation, resulting in cell cycle arrest and fork recovery. Inhibition of ATR abrogates this repair mechanism, leading to toxic DNA damage [30,80,81]; (**D**) Moreover, PARP-mediated PARylation of the multifunctional DNA stress responder TonEBP signals PCNA ubiquitination, which recruits DNA repair proteins to the site of DNA damage. Inhibition of PARP blocks TonEBP-parylation and abrogates subsequent protection against R-loop accumulation [112]. PARP (DNA repair protein): poly(ADP-ribo)polymerase; TonEBP (transcription factor): Tonicity-responsive enhancer-binding protein; PCNA (DNA replication and repair factor): proliferating cell nuclear antigen; TOP1 (resolves DNA supercoiling): topoisomerase 1; ATR (DNA damage sensor): Ataxia telangiectasia-related; ChK1: checkpoint 1; G1: growth phase 1; S: synthesis phase; G2: growth phase 2; M: mitosis.

G quadruplexes (G4) are nucleic acid structures that form primarily in GC-rich sequences. Repetitive G sequences induce the formation of a planar “G-tetrad” that can stack on each other, forming a helical structure (Figure 6A). Notably, G quadruplexes can stabilize regulatory R-loops when formed on the displaced ssDNA (reviewed in [106,107]). Small molecules that bind G quadruplexes (G4 binders) have shown great promise in cancer treatment due to their cytotoxicity [107,108]. Monohydrazone-based G4 binders induce cancer cell death by accumulating G4 and R-loops in the genome of cancer cells [109]. In *BRCA2*-mutant cancer cells, G4 binders induced R-loop accumulation followed by double-strand break and micronuclei aggregation [110]. It is worth noting that G4 binders may provide therapeutic benefits to a broad spectrum of malignant diseases due to their general mode of action. An analysis of 22 patient-derived breast cancer xenografts showed that G4-forming sequences are enriched at promoters of highly expressed genes, which leaves highly proliferating tumor cells vulnerable to G4 binders [111].

Histone deacetylase (HDAC) inhibitors such as romidepsin are clinically approved for the treatment of T cell lymphomas and multiple myelomas but are less efficacious against solid tumors. Romidepsin induces histone hyperacetylation, which leads to more open chromatin at which R-loops accumulate [113]. This, in turn, threatens the genome integrity, which is rescued by the upregulation of several DNA repair enzymes, including PARP1 [113]. Notably, administration of the PARP inhibitor Olaparib potentiated R-loop dependent DNA damage leading to increased double-strand break and decreased cell viability [113]. Therefore, HDAC inhibitor activity could be potentiated in solid tumors by combination with inhibitors of DNA damage, such as PARP inhibitors, to provide synergistic cytotoxic effects through R-loop build-up.

## 5. R-Loops as Targets for Anticancer Drugs to Combat Chemoresistance

Resistance to anticancer drugs has been described as “molecular chess”, which reflects the evasiveness of cancer cells to both classical chemotherapies (which include broadly DNA damaging agents) as well as newer targeted therapeutics [114]. It is estimated that out of every 10 cancer deaths, 9 will be attributed to anticancer drug resistance [115,116], highlighting the importance of circumventing anticancer drug resistance. One prominent mechanism of resistance is the upregulation of DNA repair mechanisms—in consequence, inhibition of more than one DNA repair pathway confers synthetic lethality (Figure 6B–D). Inhibiting the resolution of R-loops is therefore an alternative pathway that can promote efficaciousness in known anticancer drugs.

### 5.1. Inhibition of R-Loop Unwinding

Topoisomerase I TOP1 inhibitors, such as camptothecin, are approved chemotherapeutic agents used for the treatment of solid tumors [117]. Resistance against TOP1 inhibitors remains a challenge in clinical settings [118,119]. TOP1 aids in the resolution of R-loops, particularly at transcription termination sites [29]. Hepatoma cells resistant to the TOP1 inhibitor camptothecin showed an upregulation of the DNA repair protein PARP, which initiates a pathway to promote R-loop resolution [112]. PARP can thereby rescue cells from camptothecin-induced cell death [112], suggesting a clinical benefit to combination therapy to evade chemoresistance to TOP1 inhibitors.

Fast-growing solid tumors frequently experience a lack of oxygen (hypoxia) due to limited blood supply. Hypoxia is, in turn, associated with chemotherapy and radiotherapy resistance [120]. Under hypoxic conditions, cancer cells experience an increase in R-loops formation, likely due to transcriptional stress, and upregulate the expression of the R-loop resolving helicase Senataxin [121]. Interestingly, the expression of *SETX* was controlled through the unfolded protein response and the main regulator of the cellular integrated stress response, the transcription factor ATF4 [121]. Knockdown of *SETX* in hypoxic cells led to the persistence of co-transcriptional R-loops, resulting in lower replication rates and apoptosis [121]. Selective inhibition of Senataxin in hypoxic cancer cells might therefore provide an effective strategy.

The recent discovery of other R-loop-interacting proteins, many of which drive R-loop resolution [122,123], will increase the list of proteins that can be targeted to curb cancer progression through the induction of DNA damage.

### 5.2. Inhibition of R-Loop Cleavage

*BRCA2* mutated ovarian cancer cells that resist platinum chemotherapy revealed yet another mechanism that involves R-loop interacting proteins. These cells overexpress the microRNA miR-493-5P, which downregulates several R-loop processing genes [124]. These include an RNAse H, which cleaves the RNA in R-loops directly, and Flap Structure-Specific Endonuclease 1 (FEN1), which cleaves trinucleotide repeats in R-loops, resulting in overall R-loop build-up [124]. Notably, miR-493-5P also decreased Mre-11 activity, which not only impairs homologous recombination but may further induce R-loop accrual, as discussed earlier in triple-negative breast cancer cells [92]. 

RNAse H1 and RNAse H2 function differently from each other. RNAse H1 is the major nuclease to resolve R-loop-induced cell stress regardless of the cell cycle, while RNAse H2 activity has a housekeeping function post replication to avoid the persistence of R-loops [125]. RNAse H1 does not induce double-stranded nicks, while RNAse H2 cleavage sites necessitate repair [125]. In consequence, the reduction of RNAse H2 led to cell cycle arrest and double-strand breaks in leukemia cells directly while also sensitizing cancer cells to radiation and other DNA damage-inducing agents [126]. 

## 6. Conclusions

In summary, we discussed various aspects of R-loops in cancer, specifically focusing on their interplay with DNA repair proteins such as BRCA1, BRCA2, and ATR. We also explored the consequences of perturbed R-loop homeostasis in cancer cells and how it facilitates mutagenicity while also endangering proliferation and survival. The resulting genomic instability of excess R-loops can be used to induce cancer cell death and highlights possible combination strategies as treatment options.

Whether R-loop formation is beneficial or detrimental in cancer is therefore highly context-dependent. Mutations that predispose patients to increased R-loop formation or a defect in their resolution are strongly correlated with a higher risk of cancer due to the accelerated accumulation of mutations following R-loop formation. Later, once tumors are established, and both proliferation and DNA repair mechanisms are out of control, increased R-loop formation leaves cancer cells more susceptible to DNA damage too severe to support continuous cell proliferation. Inducing R-loops and/or hampering their resolution can induce synthetic lethality between inhibitors, which is especially critical in cancers with elevated R-loop appearance due to their underlying genomic instability. Manipulating the R-loops and their interacting proteins is thus a central mechanism to impair the DNA damage response and produce intolerable genomic instability.

Due to their unavoidable nature as a product of mRNA transcription, R-loops activate various proteins from different DNA repair pathways to preserve genomic and chromosomal integrity. These conserved mechanisms also highlight the R-loop-machinery as a target across clinically and histologically different cancer subtypes. Breast cancers (and likely other cancers) share an R-loop-dependent pathology, regardless of their hormone status [63,89,124,127]. In combination with available anticancer therapies that induce genomic instability, disrupt chromatin, or inhibit DNA repair pathways, these would accelerate R-loop-dependent DNA damage and cause apoptosis in fast-dividing cells. These drug candidates, which would synergically induce R-loops with known anticancer drugs, include molecules with an unusual target, such as G quadruplexes and the RNA processing machinery. Further exploration into the commonalities of R-loops homeostasis across cancers will unveil other approaches to tackling cancer through the unavoidable formation of and by aiding the persistence of R-loops.

## Data Availability

Not applicable.

## References

[1] Petermann E., Lan L., Zou L. (2022). Sources, resolution and physiological relevance of R-loops and RNA–DNA hybrids. Nat. Rev. Mol. Cell Biol..

[2] Ginno P.A., Lim Y.W., Lott P.L., Korf I., Chédin F. (2013). GC skew at the 5′ and 3′ ends of human genes links R-loop formation to epigenetic regulation and transcription termination. Genome Res..

[3] Niehrs C., Luke B. (2020). Regulatory R-loops as facilitators of gene expression and genome stability. Nat. Rev. Mol. Cell Biol..

[4] Li X., Manley J.L. (2005). Inactivation of the SR Protein Splicing Factor ASF/SF2 Results in Genomic Instability. Cell.

[5] Arora R., Lee Y., Wischnewski H., Brun C.M., Schwarz T., Azzalin C.M. (2014). RNaseH1 regulates TERRA-telomeric DNA hybrids and telomere maintenance in ALT tumour cells. Nat. Commun..

[6] Crossley M.P., Song C., Bocek M.J., Choi J.-H., Kousorous J., Sathirachinda A., Lin C., Brickner J.R., Bai G., Lans H. (2022). R-loop-derived cytoplasmic RNA–DNA hybrids activate an immune response. Nature.

[7] Brickner J.R., Garzon J.L., Cimprich K.A. (2022). Walking a tightrope: The complex balancing act of R-loops in genome stability. Mol. Cell.

[8] Santos-Pereira J.M., Aguilera A. (2015). R loops: New modulators of genome dynamics and function. Nat. Rev. Genet..

[9] Rutkauskas M., Sinkunas T., Songailiene I., Tikhomirova M., Siksnys V., Seidel R. (2015). Directional R-Loop Formation by the CRISPR-Cas Surveillance Complex Cascade Provides Efficient Off-Target Site Rejection. Cell Rep..

[10] Tuminauskaite D., Norkunaite D., Fiodorovaite M., Tumas S., Songailiene I., Tamulaitiene G., Sinkunas T. (2020). DNA interference is controlled by R-loop length in a type I-F1 CRISPR-Cas system. BMC Biol..

[11] El Hage A., Webb S., Kerr A., Tollervey D. (2014). Genome-Wide Distribution of RNA-DNA Hybrids Identifies RNase H Targets in tRNA Genes, Retrotransposons and Mitochondria. PLoS Genet..

[12] Wahba L., Costantino L., Tan F.J., Zimmer A., Koshland D. (2016). S1-DRIP-seq identifies high expression and polyA tracts as major contributors to R-loop formation. Genes Dev..

[13] Sharifi S., Bierhoff H. (2018). Regulation of RNA Polymerase I Transcription in Development, Disease, and Aging. Annu. Rev. Biochem..

[14] White R.J. (2011). Transcription by RNA polymerase III: More complex than we thought. Nat. Rev. Genet..

[15] Chen L., Chen J.-Y., Zhang X., Gu Y., Xiao R., Shao C., Tang P., Qian H., Luo D., Li H. (2017). R-ChIP Using Inactive RNase H Reveals Dynamic Coupling of R-loops with Transcriptional Pausing at Gene Promoters. Mol. Cell.

[16] Crossley M.P., Bocek M., Cimprich K.A. (2019). R-Loops as Cellular Regulators and Genomic Threats. Mol. Cell.

[17] Hou J., Liu X., Liu J. (2012). Detection of Single Nucleotide Polymorphism by RNase H-Cleavage Mediated Allele-Specific Extension Method. Biotechnol. Biotechnol. Equip..

[18] Cerritelli S.M., Sakhuja K., Crouch R.J. (2022). RNase H1, the Gold Standard for R-Loop Detection.

[19] Skourti-Stathaki K., Proudfoot N.J., Gromak N. (2011). Human Senataxin Resolves RNA/DNA Hybrids Formed at Transcriptional Pause Sites to Promote Xrn2-Dependent Termination. Mol. Cell.

[20] Bader A.S., Luessing J., Hawley B.R., Skalka G.L., Lu W.-T., Lowndes N.F., Bushell M. (2022). DDX17 Is Required for Efficient DSB Repair at DNA:RNA Hybrid Deficient Loci. Nucleic Acids Res..

[21] Khan E.S., Danckwardt S. (2022). Pathophysiological Role and Diagnostic Potential of R-Loops in Cancer and Beyond. Genes.

[22] Sollier J., Cimprich K.A. (2015). Breaking bad: R-loops and genome integrity. Trends Cell Biol..

[23] Germain C.S., Zhao H., Barlow J.H. (2021). Transcription-Replication Collisions—A Series of Unfortunate Events. Biomolecules.

[24] Chédin F. (2016). Nascent Connections: R-Loops and Chromatin Patterning. Trends Genet..

[25] Abakir A., Giles T.C., Cristini A., Foster J.M., Dai N., Starczak M., Rubio-Roldan A., Li M., Eleftheriou M., Crutchley J. (2020). N6-methyladenosine regulates the stability of RNA:DNA hybrids in human cells. Nat. Genet..

[26] Perego M.G.L., Taiana M., Bresolin N., Comi G.P., Corti S. (2019). R-Loops in Motor Neuron Diseases. Mol. Neurobiol..

[27] Richard P., Manley J.L. (2017). R Loops and Links to Human Disease. J. Mol. Biol..

[28] Wells J.P., White J., Stirling P.C. (2019). R Loops and Their Composite Cancer Connections. Trends Cancer.

[29] Promonet A., Padioleau I., Liu Y., Sanz L., Biernacka A., Schmitz A.-L., Skrzypczak M., Sarrazin A., Mettling C., Rowicka M. (2020). Topoisomerase 1 prevents replication stress at R-loop-enriched transcription termination sites. Nat. Commun..

[30] Nguyen H.D., Leong W.Y., Li W., Reddy P.N., Sullivan J.D., Walter M.J., Zou L., Graubert T.A. (2018). Spliceosome Mutations Induce R Loop-Associated Sensitivity to ATR Inhibition in Myelodysplastic Syndromes. Cancer Res..

[31] Chen L., Chen J.-Y., Huang Y.-J., Gu Y., Qiu J., Qian H., Shao C., Zhang X., Hu J., Li H. (2018). The Augmented R-Loop Is a Unifying Mechanism for Myelodysplastic Syndromes Induced by High-Risk Splicing Factor Mutations. Mol. Cell.

[32] Hatchi E., Skourti-Stathaki K., Ventz S., Pinello L., Yen A., Kamieniarz-Gdula K., Dimitrov S., Pathania S., McKinney K.M., Eaton M.L. (2015). BRCA1 Recruitment to Transcriptional Pause Sites Is Required for R-Loop-Driven DNA Damage Repair. Mol. Cell.

[33] Zhang X., Chiang H.-C., Wang Y., Zhang C., Smith S., Zhao X., Nair S.J., Michalek J., Jatoi I., Lautner M. (2017). Attenuation of RNA polymerase II pausing mitigates BRCA1-associated R-loop accumulation and tumorigenesis. Nat. Commun..

[34] Shivji M.K., Renaudin X., Williams H., Venkitaraman A.R. (2018). BRCA2 Regulates Transcription Elongation by RNA Polymerase II to Prevent R-Loop Accumulation. Cell Rep..

[35] Howlader N., Noone A., Krapcho M., Miller D., Brest A., Yu M., Ruhl J., Tatalovich Z., MAriotto A., Lewis D. (2020). SEER Cancer Statistics Review, 1975–2017.

[36] Kuchenbaecker K.B., Hopper J.L., Barnes D.R., Phillips K.-A., Mooij T.M., Roos-Blom M.-J., Jervis S., Van Leeuwen F.E., Milne R.L., Andrieu N. (2017). Risks of Breast, Ovarian, and Contralateral Breast Cancer for BRCA1 and BRCA2 Mutation Carriers. JAMA.

[37] Roy R., Chun J., Powell S.N. (2011). BRCA1 and BRCA2: Different roles in a common pathway of genome protection. Nat. Rev. Cancer.

[38] Racca C., Britton S., Hédouin S., Francastel C., Calsou P., Larminat F. (2021). BRCA1 prevents R-loop-associated centromeric instability. Cell Death Dis..

[39] Bhatia V., Barroso S.I., García-Rubio M.L., Tumini E., Herrera-Moyano E., Aguilera A. (2014). BRCA2 prevents R-loop accumulation and associates with TREX-2 mRNA export factor PCID2. Nature.

[40] Skourti-Stathaki K., Proudfoot N.J. (2014). A double-edged sword: R loops as threats to genome integrity and powerful regulators of gene expression. Genes Dev..

[41] Hashizume R., Fukuda M., Maeda I., Nishikawa H., Oyake D., Yabuki Y., Ogata H., Ohta T. (2001). The RING Heterodimer BRCA1-BARD1 Is a Ubiquitin Ligase Inactivated by a Breast Cancer-derived Mutation. J. Biol. Chem..

[42] Yu X., Chini C.C.S., He M., Mer G., Chen J. (2003). The BRCT Domain Is a Phospho-Protein Binding Domain. Science.

[43] Prakash R., Zhang Y., Feng W., Jasin M. (2015). Homologous Recombination and Human Health: The Roles of BRCA1, BRCA2, and Associated Proteins. Cold Spring Harb. Perspect. Biol..

[44] Sharan S.K., Morimatsu M., Albrecht U., Lim D.-S., Regel E., Dinh C., Sands A., Eichele G., Hasty P., Bradley A. (1997). Embryonic lethality and radiation hypersensitivity mediated by Rad51 in mice lacking Brca2. Nature.

[45] Zheng L., Li S., Boyer T.G., Lee W.-H. (2000). Lessons learned from BRCA1 and BRCA2. Oncogene.

[46] Bhat K.P., Cortez D. (2018). RPA and RAD51: Fork reversal, fork protection, and genome stability. Nat. Struct. Mol. Biol..

[47] Schlacher K., Christ N., Siaud N., Egashira A., Wu H., Jasin M. (2011). Double-Strand Break Repair-Independent Role for BRCA2 in Blocking Stalled Replication Fork Degradation by MRE11. Cell.

[48] Szabo C.I., Worley T., Monteiro A.N. (2004). Understanding Germ-Line Mutations in BRCA1. Cancer Biol. Ther..

[49] Alonso M.S.M., Noordermeer S.M. (2021). Untangling the crosstalk between BRCA1 and R-loops during DNA repair. Nucleic Acids Res..

[50] Hopkins J.L., Lan L., Zou L. (2022). DNA repair defects in cancer and therapeutic opportunities. Genes Dev..

[51] McKinley K., Cheeseman I.M. (2016). The molecular basis for centromere identity and function. Nat. Rev. Mol. Cell Biol..

[52] Adelman K., Lis J.T. (2012). Promoter-proximal pausing of RNA polymerase II: Emerging roles in metazoans. Nat. Rev. Genet..

[53] Zhou Q., Li T., Price D.H. (2012). RNA Polymerase II Elongation Control. Annu. Rev. Biochem..

[54] Buckley N., Tsaoir C.B.N.A., Blayney J.K., Oram L.C., Crawford N.T., D’Costa Z.C., Quinn J.E., Kennedy R.D., Harkin D.P., Mullan P.B. (2013). BRCA1 is a key regulator of breast differentiation through activation of Notch signalling with implications for anti-endocrine treatment of breast cancers. Nucleic Acids Res..

[55] Furuta S., Jiang X., Gu B., Cheng E., Chen P.-L., Lee W.-H. (2005). Depletion of BRCA1 impairs differentiation but enhances proliferation of mammary epithelial cells. Proc. Natl. Acad. Sci. USA.

[56] Chiang H.-C., Chiang H.-C., Zhang X., Zhang X., Li J., Li J., Zhao X., Zhao X., Chen J., Chen J. (2019). BRCA1-associated R-loop affects transcription and differentiation in breast luminal epithelial cells. Nucleic Acids Res..

[57] Gaspar T.B., Sá A., Lopes J.M., Sobrinho-Simões M., Soares P., Vinagre J. (2018). Telomere Maintenance Mechanisms in Cancer. Genes.

[58] Bejarano L., Bosso G., Louzame J., Serrano R., Gómez-Casero E., Martinez-Torrecuadrada J.L., Martínez S., Blanco-Aparicio C., Pastor J., A Blasco M. (2019). Multiple cancer pathways regulate telomere protection. EMBO Mol. Med..

[59] Feretzaki M., Pospisilova M., Fernandes R.V., Lunardi T., Krejci L., Lingner J. (2020). RAD51-dependent recruitment of TERRA lncRNA to telomeres through R-loops. Nature.

[60] Vohhodina J., Goehring L.J., Liu B., Kong Q., Botchkarev V.V., Huynh M., Liu Z., Abderazzaq F.O., Clark A.P., Ficarro S.B. (2021). BRCA1 binds TERRA RNA and suppresses R-Loop-based telomeric DNA damage. Nat. Commun..

[61] Pompili L., Maresca C., Stritto A.D., Biroccio A., Salvati E. (2019). BRCA2 Deletion Induces Alternative Lengthening of Telomeres in Telomerase Positive Colon Cancer Cells. Genes.

[62] Shiromoto Y., Sakurai M., Minakuchi M., Ariyoshi K., Nishikura K. (2021). ADAR1 RNA editing enzyme regulates R-loop formation and genome stability at telomeres in cancer cells. Nat. Commun..

[63] Stork C.T., Bocek M., Crossley M.P., Sollier J., A Sanz L., Chédin F., Swigut T., A Cimprich K. (2016). Co-transcriptional R-loops are the main cause of estrogen-induced DNA damage. eLife.

[64] Holt I.J. (2022). R-Loops and Mitochondrial DNA Metabolism. Methods Mol. Biol..

[65] Xu B., Clayton D.A. (1996). RNA-DNA hybrid formation at the human mitochondrial heavy-strand origin ceases at replication start sites: An implication for RNA-DNA hybrids serving as primers. EMBO J..

[66] Szczesny R.J., Borowski L.S., Malecki M., Wojcik M.A., Stepien P.P., Golik P. (2012). RNA Degradation in Yeast and Human Mitochondria. Biochim. Biophys. Acta (BBA) Gene Regul. Mech..

[67] Silva S., Camino L.P., Aguilera A. (2018). Human mitochondrial degradosome prevents harmful mitochondrial R loops and mitochondrial genome instability. Proc. Natl. Acad. Sci. USA.

[68] Holmes J.B., Akman G., Wood S.R., Sakhuja K., Cerritelli S.M., Moss C., Bowmaker M.R., Jacobs H.T., Crouch R.J., Holt I.J. (2015). Primer retention owing to the absence of RNase H1 is catastrophic for mitochondrial DNA replication. Proc. Natl. Acad. Sci. USA.

[69] Boland M.L., Chourasia A.H., MacLeod K.F. (2013). Mitochondrial Dysfunction in Cancer. Front. Oncol..

[70] Yi Y.W., Kang H.J., Bae I. (2014). BRCA1 and Oxidative Stress. Cancers.

[71] Renaudin X., Lee M., Shehata M., Surmann E.-M., Venkitaraman A.R. (2021). BRCA2 deficiency reveals that oxidative stress impairs RNaseH1 function to cripple mitochondrial DNA maintenance. Cell Rep..

[72] Lecona E., Fernandez-Capetillo O. (2018). Targeting ATR in cancer. Nat. Rev. Cancer.

[73] Jo U., Senatorov I.S., Zimmermann A., Saha L.K., Murai Y., Kim S.H., Rajapakse V.N., Elloumi F., Takahashi N., Schultz C.W. (2021). Novel and Highly Potent ATR Inhibitor M4344 Kills Cancer Cells with Replication Stress, and Enhances the Chemotherapeutic Activity of Widely Used DNA Damaging Agents. Mol. Cancer Ther..

[74] Plummer R., Dean E., Arkenau H.-T., Redfern C., Spira A.I., Melear J.M., Chung K.Y., Ferrer-Playan J., Goddemeier T., Locatelli G. (2022). A phase 1b study evaluating the safety and preliminary efficacy of berzosertib in combination with gemcitabine in patients with advanced non-small cell lung cancer. Lung Cancer.

[75] Kwon M., Kim G., Kim R., Kim K.-T., Kim S.T., Smith S., Mortimer P.G.S., Hong J.Y., Loembé A.-B., Irurzun-Arana I. (2022). Phase II study of ceralasertib (AZD6738) in combination with durvalumab in patients with advanced gastric cancer. J. Immunother. Cancer.

[76] Middleton M.R., Dean E., Evans T.R.J., Shapiro G.I., Pollard J., Hendriks B.S., Falk M., Diaz-Padilla I., Plummer R. (2021). Phase 1 study of the ATR inhibitor berzosertib (formerly M6620, VX-970) combined with gemcitabine ± cisplatin in patients with advanced solid tumours. Br. J. Cancer.

[77] Matos D.A., Zhang J.-M., Ouyang J., Nguyen H.D., Genois M.-M., Zou L. (2020). ATR Protects the Genome against R Loops through a MUS81-Triggered Feedback Loop. Mol. Cell.

[78] Wang T., Zhang P., Li C., Liu W., Shen Q., Yang L., Xie G., Bai J., Li R., Tao K. (2022). MUS81 Inhibition Enhances the Anticancer Efficacy of Talazoparib by Impairing ATR/CHK1 Signaling Pathway in Gastric Cancer. Front. Oncol..

[79] Kabeche L., Nguyen H.D., Buisson R., Zou L. (2018). A mitosis-specific and R loop–driven ATR pathway promotes faithful chromosome segregation. Science.

[80] Gorthi A., Romero J.C., Loranc E., Cao L., Lawrence L.A., Goodale E., Iniguez A.B., Bernard X., Masamsetti V.P., Roston S. (2018). EWS–FLI1 increases transcription to cause R-loops and block BRCA1 repair in Ewing sarcoma. Nature.

[81] Chabanon R.M., Morel D., Eychenne T., Colmet-Daage L., Bajrami I., Dorvault N., Garrido M., Meisenberg C., Lamb A., Ngo C. (2021). PBRM1 Deficiency Confers Synthetic Lethality to DNA Repair Inhibitors in Cancer. Cancer Res..

[82] Paronetto M.P. (2013). Ewing Sarcoma Protein: A Key Player in Human Cancer. Int. J. Cell Biol..

[83] Abraham K.J., Khosraviani N., Chan J.N.Y., Gorthi A., Samman A., Zhao D.Y., Wang M., Bokros M., Vidya E., Ostrowski L.A. (2020). Nucleolar RNA polymerase II drives ribosome biogenesis. Nature.

[84] Kanellis D.C., Espinoza J.A., Zisi A., Sakkas E., Bartkova J., Katsori A.-M., Boström J., Dyrskjøt L., Broholm H., Altun M. (2021). The exon-junction complex helicase eIF4A3 controls cell fate via coordinated regulation of ribosome biogenesis and translational output. Sci. Adv..

[85] Lindström M.S., Bartek J., Maya-Mendoza A. (2022). P53 at the crossroad of DNA replication and ribosome biogenesis stress pathways. Cell Death Differ..

[86] Moch H., Cubilla A.L., Humphrey P.A., Reuter V.E., Ulbright T.M. (2016). The 2016 WHO Classification of Tumours of the Urinary System and Male Genital Organs—Part A: Renal, Penile, and Testicular Tumours. Eur. Urol..

[87] Brownlee P.M., Chambers A.L., Cloney R., Bianchi A., Downs J.A. (2014). BAF180 Promotes Cohesion and Prevents Genome Instability and Aneuploidy. Cell Rep..

[88] Krais J.J., Wang Y., Patel P., Basu J., Bernhardy A.J., Johnson N. (2021). RNF168-mediated localization of BARD1 recruits the BRCA1-PALB2 complex to DNA damage. Nat. Commun..

[89] Patel P.S., Abraham K.J., Guturi K.K.N., Halaby M.-J., Khan Z., Palomero L., Ho B., Duan S., St-Germain J., Algouneh A. (2021). RNF168 regulates R-loop resolution and genomic stability in BRCA1/2-deficient tumors. J. Clin. Investig..

[90] Lam F.C., Kong Y.W., Huang Q., Han T.-L.V., Maffa A.D., Kasper E.M., Yaffe M.B. (2020). BRD4 prevents the accumulation of R-loops and protects against transcription–replication collision events and DNA damage. Nat. Commun..

[91] Edwards D.S., Maganti R., Tanksley J.P., Luo J., Park J.J., Balkanska-Sinclair E., Ling J., Floyd S.R. (2020). BRD4 Prevents R-Loop Formation and Transcription-Replication Conflicts by Ensuring Efficient Transcription Elongation. Cell Rep..

[92] Fagan-Solis K.D., Simpson D.A., Kumar R.J., Martelotto L.G., Mose L.E., Rashid N.U., Ho A.Y., Powell S.N., Wen Y.H., Parker J.S. (2020). A P53-Independent DNA Damage Response Suppresses Oncogenic Proliferation and Genome Instability. Cell Rep..

[93] Bai X., Ni J., Beretov J., Graham P., Li Y. (2021). Triple-negative breast cancer therapeutic resistance: Where is the Achilles’ heel?. Cancer Lett..

[94] Wang Y.-Y., Hung A.C., Lo S., Hsieh Y.-C., Yuan S.-S.F. (2021). MRE11 as a molecular signature and therapeutic target for cancer treatment with radiotherapy. Cancer Lett..

[95] Lambo S., Gröbner S.N., Rausch T., Waszak S.M., Schmidt C., Gorthi A., Romero J.C., Mauermann M., Brabetz S., Krausert S. (2019). The molecular landscape of ETMR at diagnosis and relapse. Nature.

[96] Lu W.-T., Hawley B.R., Skalka G.L., Baldock R.A., Smith E.M., Bader A.S., Malewicz M., Watts F.Z., Wilczynska A., Bushell M. (2018). Drosha drives the formation of DNA:RNA hybrids around DNA break sites to facilitate DNA repair. Nat. Commun..

[97] Smolinski D. (2022). R-loops at microRNA encoding loci promote co-transcriptional processing of pri-miRNAs in plants. Nat. Plants.

[98] Itzen F., Greifenberg A.K., Bösken C.A., Geyer M. (2014). Brd4 activates P-TEFb for RNA polymerase II CTD phosphorylation. Nucleic Acids Res..

[99] Muhar M., Ebert A., Neumann T., Umkehrer C., Jude J., Wieshofer C., Rescheneder P., Lipp J.J., Herzog V.A., Reichholf B. (2018). SLAM-seq defines direct gene-regulatory functions of the BRD4-MYC axis. Science.

[100] Filippakopoulos P., Qi J., Picaud S., Shen Y., Smith W.B., Fedorov O., Morse E.M., Keates T., Hickman T.T., Felletar I. (2010). Selective inhibition of BET bromodomains. Nature.

[101] Dawson M.A., Prinjha R.K., Dittmann A., Giotopoulos G., Bantscheff M., Chan W.-I., Robson S.C., Chung C.-W., Hopf C., Savitski M.M. (2011). Inhibition of BET recruitment to chromatin as an effective treatment for MLL-fusion leukaemia. Nature.

[102] Nicodeme E., Jeffrey K.L., Schaefer U., Beinke S., Dewell S., Chung C.-W., Chandwani R., Marazzi I., Wilson P., Coste H. (2010). Suppression of inflammation by a synthetic histone mimic. Nature.

[103] Tajima N., Fukui K., Uesato N., Maruhashi J., Yoshida T., Watanabe Y., Tojo A. (2010). JTE-607, a multiple cytokine production inhibitor, induces apoptosis accompanied by an increase in p21^waf1/cip1^ in acute myelogenous leukemia cells. Cancer Sci..

[104] Uesato N., Fukui K., Maruhashi J., Tojo A., Tajima N. (2006). JTE-607, a multiple cytokine production inhibitor, ameliorates disease in a SCID mouse xenograft acute myeloid leukemia model. Exp. Hematol..

[105] Ross N.T., Lohmann F., Carbonneau S., Fazal A., Weihofen W.A., Gleim S., Salcius M., Sigoillot F., Henault M., Carl S.H. (2020). CPSF3-dependent pre-mRNA processing as a druggable node in AML and Ewing’s sarcoma. Nat. Chem. Biol..

[106] Miglietta G., Russo M., Capranico G. (2020). G-quadruplex–R-loop interactions and the mechanism of anticancer G-quadruplex binders. Nucleic Acids Res..

[107] Camarillo R., Jimeno S., Huertas P. (2021). The Effect of Atypical Nucleic Acids Structures in DNA Double Strand Break Repair: A Tale of R-loops and G-Quadruplexes. Front. Genet..

[108] Kosiol N., Juranek S., Brossart P., Heine A., Paeschke K. (2021). G-quadruplexes: A promising target for cancer therapy. Mol. Cancer.

[109] Amato J., Miglietta G., Morigi R., Iaccarino N., Locatelli A., Leoni A., Novellino E., Pagano B., Capranico G., Randazzo A. (2020). Monohydrazone Based G-Quadruplex Selective Ligands Induce DNA Damage and Genome Instability in Human Cancer Cells. J. Med. Chem..

[110] De Magis A., Manzo S.G., Russo M., Marinello J., Morigi R., Sordet O., Capranico G. (2019). DNA damage and genome instability by G-quadruplex ligands are mediated by R loops in human cancer cells. Proc. Natl. Acad. Sci. USA.

[111] Haensel-Hertsch R., Simeone A., Shea A., Hui W.W.I., Zyner K.G., Marsico G., Rueda O.M., Bruna A., Martin A., Zhang X. (2020). Landscape of G-quadruplex DNA structural regions in breast cancer. Nat. Genet..

[112] Ye B.J., Kang H.J., Lee-Kwon W., Kwon H.M., Choi S.Y. (2021). PARP1-mediated PARylation of TonEBP prevents R-loop–associated DNA damage. DNA Repair.

[113] Safari M., Litman T., Robey R.W., Aguilera A., Chakraborty A.R., Reinhold W.C., Basseville A., Petrukhin L., Scotto L., O’Connor O.A. (2021). R-Loop–Mediated ssDNA Breaks Accumulate Following Short-Term Exposure to the HDAC Inhibitor Romidepsin. Mol. Cancer Res..

[114] Cree I.A., Charlton P. (2017). Molecular chess? Hallmarks of anti-cancer drug resistance. BMC Cancer.

[115] Bukowski K., Kciuk M., Kontek R. (2020). Mechanisms of Multidrug Resistance in Cancer Chemotherapy. Int. J. Mol. Sci..

[116] Wang X., Zhang H., Chen X. (2019). Drug resistance and combating drug resistance in cancer. Cancer Drug Resist..

[117] Moukharskaya J., Verschraegen C. (2012). Topoisomerase 1 Inhibitors and Cancer Therapy. Hematol. Clin. N. Am..

[118] Ando K., Shah A.K., Sachdev V., Kleinstiver B.P., Taylor-Parker J., Welch M.M., Hu Y., Salgia R., White F.M., Parvin J.D. (2017). Camptothecin resistance is determined by the regulation of topoisomerase I degradation mediated by ubiquitin proteasome pathway. Oncotarget.

[119] Rubin E.H., Li T.-K., Duann P., Liu L.F. (1996). Cellular Resistance to Topoisomerase Poisons. Cancer Treat. Res..

[120] Hammond E., Asselin M.-C., Forster D., O’Connor J., Senra J., Williams K. (2014). The Meaning, Measurement and Modification of Hypoxia in the Laboratory and the Clinic. Clin. Oncol..

[121] Ramachandran S., Ma T.S., Griffin J., Ng N., Foskolou I.P., Hwang M.-S., Victori P., Cheng W.-C., Buffa F.M., Leszczynska K.B. (2021). Hypoxia-induced SETX links replication stress with the unfolded protein response. Nat. Commun..

[122] Kumar A., Fournier L.-A., Stirling P.C. (2022). Integrative analysis and prediction of human R-loop binding proteins. G3 Genes Genomes Genet..

[123] Yan Q., Wulfridge P., Doherty J., Fernandez-Luna J.L., Real P.J., Tang H.-Y., Sarma K. (2022). Proximity labeling identifies a repertoire of site-specific R-loop modulators. Nat. Commun..

[124] Meghani K., Fuchs W., Detappe A., Drané P., Gogola E., Rottenberg S., Jonkers J., Matulonis U., Swisher E.M., Konstantinopoulos P.A. (2018). Multifaceted Impact of MicroRNA 493-5p on Genome-Stabilizing Pathways Induces Platinum and PARP Inhibitor Resistance in BRCA2-Mutated Carcinomas. Cell Rep..

[125] Lockhart A., Pires V.B., Bento F., Kellner V., Luke-Glaser S., Yakoub G., Ulrich H.D., Luke B. (2019). RNase H1 and H2 Are Differentially Regulated to Process RNA-DNA Hybrids. Cell Rep..

[126] Ghosh D., Kumari S., Raghavan S.C. (2022). Depletion of RNASEH2 Activity Leads to Accumulation of DNA Double-strand Breaks and Reduced Cellular Survivability in T Cell Leukemia. J. Mol. Biol..

[127] Tu X., Kahila M.M., Zhou Q., Yu J., Kalari K.R., Wang L., Harmsen W.S., Yuan J., Boughey J.C., Matthew P. (2018). ATR Inhibition Is a Promising Radiosensitizing Strategy for Triple-Negative Breast Cancer. Mol. Cancer Ther..

